# Flow cytometric evaluation of the neutrophil compartment in COVID-19 at hospital presentation: A normal response to an abnormal situation

**DOI:** 10.1002/JLB.5COVA0820-520RRR

**Published:** 2020-12-22

**Authors:** Roy Spijkerman, Suzanne H Bongers, Bas J J Bindels, Gerjen H Tinnevelt, Giulio Giustarini, Nikita K N Jorritsma, Wiebe Buitenwerf, Daan E J van Spengler, Eveline M Delemarre, Stefan Nierkens, Harriët M R van Goor, Jeroen J Jansen, Nienke Vrisekoop, Falco Hietbrink, Luke P H Leenen, Karin A H Kaasjager, Leo Koenderman, the COVPACH study group

**Affiliations:** Department of Respiratory Medicine, University Medical Center Utrecht, Heidelberglaan, Utrecht, The Netherlands; Department of Trauma Surgery, University Medical Center Utrecht, Heidelberglaan, Utrecht, The Netherlands; Center for Translational Immunology (CTI), University Medical Center Utrecht, Heidelberglaan, Utrecht, The Netherlands; Department of Trauma Surgery, University Medical Center Utrecht, Heidelberglaan, Utrecht, The Netherlands; Center for Translational Immunology (CTI), University Medical Center Utrecht, Heidelberglaan, Utrecht, The Netherlands; Department of Respiratory Medicine, University Medical Center Utrecht, Heidelberglaan, Utrecht, The Netherlands; Center for Translational Immunology (CTI), University Medical Center Utrecht, Heidelberglaan, Utrecht, The Netherlands; Institute for Molecules and Materials, Radboud University, Heyendaalseweg, Nijmegen, The Netherlands; Department of Respiratory Medicine, University Medical Center Utrecht, Heidelberglaan, Utrecht, The Netherlands; Center for Translational Immunology (CTI), University Medical Center Utrecht, Heidelberglaan, Utrecht, The Netherlands; Department of Respiratory Medicine, University Medical Center Utrecht, Heidelberglaan, Utrecht, The Netherlands; Center for Translational Immunology (CTI), University Medical Center Utrecht, Heidelberglaan, Utrecht, The Netherlands; Department of Respiratory Medicine, University Medical Center Utrecht, Heidelberglaan, Utrecht, The Netherlands; Center for Translational Immunology (CTI), University Medical Center Utrecht, Heidelberglaan, Utrecht, The Netherlands; Department of Respiratory Medicine, University Medical Center Utrecht, Heidelberglaan, Utrecht, The Netherlands; Center for Translational Immunology (CTI), University Medical Center Utrecht, Heidelberglaan, Utrecht, The Netherlands; Center for Translational Immunology (CTI), University Medical Center Utrecht, Heidelberglaan, Utrecht, The Netherlands; Center for Translational Immunology (CTI), University Medical Center Utrecht, Heidelberglaan, Utrecht, The Netherlands; Department of Internal Medicine, University Medical Center Utrecht, Heidelberglaan, Utrecht, The Netherlands; Institute for Molecules and Materials, Radboud University, Heyendaalseweg, Nijmegen, The Netherlands; Department of Respiratory Medicine, University Medical Center Utrecht, Heidelberglaan, Utrecht, The Netherlands; Center for Translational Immunology (CTI), University Medical Center Utrecht, Heidelberglaan, Utrecht, The Netherlands; Department of Trauma Surgery, University Medical Center Utrecht, Heidelberglaan, Utrecht, The Netherlands; Department of Trauma Surgery, University Medical Center Utrecht, Heidelberglaan, Utrecht, The Netherlands; Department of Internal Medicine, University Medical Center Utrecht, Heidelberglaan, Utrecht, The Netherlands; Department of Respiratory Medicine, University Medical Center Utrecht, Heidelberglaan, Utrecht, The Netherlands; Center for Translational Immunology (CTI), University Medical Center Utrecht, Heidelberglaan, Utrecht, The Netherlands; Department of Respiratory Medicine, University Medical Center Utrecht, Heidelberglaan, Utrecht, The Netherlands

**Keywords:** activation, CD10, flow cytometry, neprilysin, neutrophil, SARS-CoV-2

## Abstract

Coronavirus disease 2019 (COVID-19) is a rapidly emerging pandemic disease caused by the severe acute respiratory syndrome coronavirus 2 (SARS-CoV-2). Critical COVID-19 is thought to be associated with a hyper-inflammatory process that can develop into acute respiratory distress syndrome, a critical disease normally mediated by dysfunctional neutrophils. This study tested the hypothesis whether the neutrophil compartment displays characteristics of hyperinflammation in COVID-19 patients. Therefore, a prospective study was performed on all patients with suspected COVID-19 presenting at the emergency room of a large academic hospital. Blood drawn within 2 d after hospital presentation was analyzed by point-of-care automated flow cytometry and compared with blood samples collected at later time points. COVID-19 patients did not exhibit neutrophilia or eosinopenia. Unexpectedly neutrophil activation markers (CD11b, CD16, CD10, and CD62L) did not differ between COVID-19-positive patients and COVID-19-negative patients diagnosed with other bacterial/viral infections, or between COVID-19 severity groups. In all patients, a decrease was found in the neutrophil maturation markers indicating an inflammation-induced left shift of the neutrophil compartment. In COVID-19 this was associated with disease severity.

## Graphical Abstract

COVID-19 is associated with a decrease in maturation markers in the neutrophil compartment without clear signs of activation at hospital presentation (figure made with the use of Biorender software).

**Figure:**
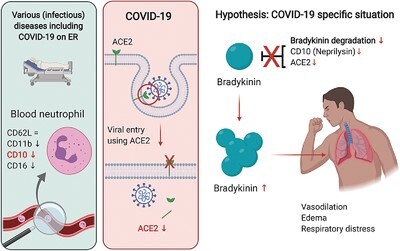

## Introduction

In December 2019, severe acute respiratory syndrome coronavirus 2 (SARS-CoV-2) emerged from Wuhan City, Hubei Province, in China.^1^ Since then, the virus has spread globally, causing a pandemic of coronavirus disease 2019 (COVID-19). The disease severity greatly varies between patients, ranging from mild complaints to intensive care unit (ICU) admittance and death. Groups at risk for severe disease are the elderly, patients with chronic diseases, and individuals suffering from obesity.^2^ Severe disease is associated with both pulmonary manifestations and, albeit less abundant, nonrespiratory symptoms.^3^ The disease can become life threatening when tissue function becomes critically compromised, such as seen during respiratory failure and acute respiratory distress syndrome (ARDS). In addition, cardiovascular complications can also lead to pathology in the intestine, heart, brain, and renal tissue.^4^ The underlying mechanism leading to tissue damage in COVID-19 is still uncertain, but a toxic combination of abnormal coagulation, systemic thrombosis, thrombo-embolisms, and hyperinflammation is thought to mediate critical disease in COVID-19 patients.^5^^,^^6^

The ARDS found in COVID-19 patients is characterized by decreased oxygenation and rapid respiratory failure.^7^ The pathophysiology of ARDS is normally mediated by malfunctioning of the neutrophil compartment leading to accumulation and specific activation of these cells in the pulmonary tissue, which in turn causes collateral damage characterized by destruction of epithelial and endothelial cells.^8–12^ Tissue damage is caused by neutrophil-driven mechanisms that are normally employed to kill microorganisms, which include production of reactive oxygen species, degranulation of toxic proteins and enzymes, and netosis.^10^^,^^13^^,^^14^ This leads to increased vascular permeability and protein-rich alveolar edema causing decreased gas exchange and hypoxemia.^15^ Many COVID-19 patients meet the Berlin definition for ARDS,^16^ but it remains to be elucidated whether ARDS seen in COVID-19 patients is similar to the archetypal ARDS seen in other patients. Although both are characterized by bilateral consolidations and severe hypoxia, COVID-19-mediated ARDS seems to have a later onset than “classical” ARDS and is more often associated with a relatively normal lung compliance.^8^^,^^17^ Also, the role of neutrophils in COVID-19-mediated ARDS is still unclear.

A variety of increased proinflammatory cytokines (TNF-α, IL-1β, IL-6, IL-8) is found in the blood of COVID-19 patients, suggesting a proinflammatory state^18^ and a hyper-activation of the immune system.^19^ Therefore, several studies have suggested that ARDS in COVID-19 is mainly caused by a cytokine storm and that anti-inflammatory drugs might be helpful.^20^ However, direct evidence that COVID-19-related ARDS is characterized by inflammation is missing.^21^ Alternatively, COVID-19-associated ARDS might be caused by pulmonary edema via a dysfunctional bradykinin metabolism. This latter explanation was put forward as the angiotensin-converting enzyme 2 (ACE2) receptor, important in bradykinin1-9 inactivation, is the main *porte d’entrée* of SARS-CoV-2 in airway epithelium and its expression is decreased upon virus entry into the epithelial cells.^22^ Interestingly, bradykinin1-9 can also be inactivated by neprilysin (CD10), which is an important activation and differentiation marker expressed by neutrophils, possibly linking neutrophils with bradykinin-induced pulmonary edema.^23^^,^^24^

The increase in expression of activation markers on neutrophils is generally used to study the role of neutrophils in vivo in health and disease.^25–27^ However, these cells are notoriously sensitive to ex vivo manipulation and it is therefore essential to minimally manipulate the cells ex vivo. We have recently shown that neutrophils become activated in the blood collection tube relatively quickly (<1 h) after venipuncture even before processing, which masks essential information about the state of the cells in vivo.^28^ Fortunately, fast analysis by 24/7 automated flow cytometry now circumvents these problems and allows fast and reproducible measurement of the activation state of human neutrophils in health and disease conditions such as COVID-19.

In order to further understand the pathophysiology of this pandemic disease, we investigated whether the neutrophil compartment is actively involved in COVID-19-associated disease in patients and if this is specific to COVID-19. Additionally, we investigated whether the neutrophil compartment shows signs of hyperinflammation during COVID-19.

## Methods

### Study design and setting

All patients with suspected COVID-19 who presented at the University Medical Center Utrecht (UMCU) during the Dutch epidemic between March 19, 2020, and May 17, 2020, were included in a prospective cohort study. The UMCU is a tertiary academic hospital, which has a regional referral function. The first blood sample was drawn at the emergency room (ER) or within 2 d on the COVID-19 ward. Longitudinal samples were also collected during routine venipuncture on the COVID-19 wards. The onset of symptoms was defined as the day that patients self-reported the onset of his/her symptoms at home. No blood samples were drawn at the ICU. All patients were tested for the presence of virus by SARS-CoV-2-specific PCR. Patients were categorized as “COVID-19 positive” if PCR results were positive. Patients who tested negative for SARS-CoV-2 were lost to follow up for this study. Baseline characteristics and clinical parameters of the included COVID-19 patients are shown in Table 1. Only patients older than 18 were included in this study. All immunocompromised patients (presence of any immunosuppressive condition or use of systemic immunosuppressive medication as per the International Classification of Disease, 9th revision^29^) were excluded from the analysis.

**TABLE 1:** Basic characteristics

	Moderate (*n* = 10)	Severe (*n* = 16)	Critical (*n* = 77)	*P* value*
Female	5 (56%)	8 (53%)	31 (42%)	0.64
Age	58.5 (53.0, 70.2)	57.0 (52.7, 71.9)	69.4 (59.1, 76.7)	0.095
BMI	26.6 (25.2, 29.2)	25.8 (25.2, 31.2)	28.6 (24.8, 31.9)	0.45
Days since disease onset	4.5 (1.0, 6.5)	7.0 (6.0, 13.0)	7.0 (4.0, 10.0)	0.13
**Clinical parameters at presentation**				
FiO_2_ (%)	21 (21, 21)	21 (21, 22)	36 (28, 44)	<0.001
Oxygen required	2 (20%)	4 (25%)	61 (79%)	<0.001
*sNose mask*	2	4	41	
*Venturi mask 40%/60%*	0	0	6	
*Nonrebreathing mask*	0	0	14	
Respiratory rate (/min)	17 (16, 18)	22 (17, 24)	24 (20, 30)	0.002
SBP (mmHg)	137 (125, 170)	134 (123, 152)	134 (119, 149)	0.69
DBP (mmHg)	78 (63, 87)	80 (62, 86)	79 (69, 85)	0.93
HR (bpm)	89 (76, 101)	80 (70, 94)	89 (79, 105)	0.075
Temperature (°C)	37.5 (37.2, 38.6)	38.1 (37.0, 38.6)	38.0 (37.2, 38.4)	0.94
CRP (g/L)	13.5 (5.5, 39.0)	54.0 (42.0, 63.0)	95.0 (34.5, 152.5)	0.003
Total leukocyte count (×10^9^/L)	5.7 (4.4, 6.4)	6.0 (4.5, 7.0)	6.8 (5.1, 10.3)	0.087
Arterial saturation	0.97 (0.97, 0.98)	0.96 (0.95, 0.98)	0.94 (0.92, 0.96)	0.061
**Clinical admission**				
Length of hospital stay (days)		5.0 (3.0, 7.5)	13.0 (6.0, 20.0)	<0.001
ICU admission		0 (0%)	22 (36%)	0.006
Mortality		1 (6%)	21 (27%)	0.072
**Samples**				
Samples	10 (100%)	10 (63%)	60 (78%)	

* *P* value using χ^2^, Kruskal-Wallis, or Mann-Whitney *U*-test where appropriate.

** Categorical values are described as *N* (%) and continuous values are described as median with interquartile range.

*** Abbreviations: BMI—body mass index; SBP—systolic blood pressure; DBP—diastolic blood pressure; CRP—C-reactive protein; and ICU—intensive care unit.

**** Number of missing values: BMI—12%; onset of symptoms—12%; respiratory rate—16%; systolic blood pressure—4%; diastolic blood pressure 5%; pulse rate—8%; temperature—11%; CRP—18%; total leukocyte count—17%; arterial saturation—43%; absolute cell counts—2%; and all others: no missing values.

### Study procedure

For standard-of-care diagnostic workup in the emergency department and/or during standard blood drawing at the COVID-19 ward, one 4 ml or 9 ml VACUETTE sodium heparin blood collection tube (Greiner Bio-One, Kremsmünster, Austria) was obtained from each patient. Thereafter, the blood tube was placed in the automated AQUIOS CL “Load & Go” flow cytometer (Beckman Coulter, Miami, FL, USA) that was located at the point of care in the emergency department. This was done as fast as possible after blood drawing, because neutrophils become easily and quickly activated ex vivo.^28^ Healthy control blood obtained from the Mini Donor Service (Mini Donor Dienst) at UMCU was analyzed in a similar manner. Healthy control subjects were chosen based on donor availability. COVID-19 measures implemented in the hospital caused a shortage of available donors and therefore these could not be matched to the study patient population for age or gender. Thus, blood from healthy controls was drawn at the out-patient clinic of UMCU, which is in close proximity to the emergency department and sample handling times were similar to those of patients included in this study. The median age of the healthy control subjects was 24 yr (interquartile range [IQR]: 23–27) 33.3% female and 66.7% male. Healthy control subjects were not on any medications for chronic diseases that could impact study results.

### Flow cytometry analysis by automated AQUIOS CL “Load & Go” flow cytometer

The AQUIOS CL combines robotic automated sample preparation with automated analysis of single cells using flow cytometry.^28^ A cassette filled with blood tubes is placed in the machine. Hereafter, the device reads the barcodes on the blood tubes, automatically mixes and pipets the blood, and proceeds with the antibody staining. After 15 min of incubation, the blood is lysed by the addition of 335 μl of lysing reagent A followed by 100 μl of lysing reagent B. Lysing reagent A is a cyanide-free lytic reagent that lyses red blood cells. Lysing reagent B slows the reaction caused by reagent A and preserves the white blood cells for measurement in the flow cell. Finally, the prepared sample is aspirated for analysis. Absolute white blood cell count is based on an electronic-volume measurement.

For this research purpose, a customized antibody cocktail was prepared and tested in the presence and absence of the bacterial/mitochondrial-derived activator N-Formyl-norleucyl-leucyl-phenylalanine (fNLF) (BioCat GmbH, Heidelberg, Germany) at an end concentration of 10^−5^ M. The antibody panel consisted of CD16-FITC (clone 3G8), CD11b-PE (clone Bear1), CD62L-ECD (clone DREG56), CD10-PC5 (clone ALB1), and CD64-PC7 (clone 22; all clones from Beckman Coulter).

### Analysis of flow cytometry data

For in-depth analysis, the .lmd data files were exported from the AQUIOS CL and imported into FlowJo analysis software (Tree Star, Inc., Ashland, OR, USA). Polymorphonuclear leukocytes were gated based on forward scatter and side scatter. Eosinophils were identified based on CD16/CD62L expression and excluded from the polymorphonuclear leukocytes gate. Then, neutrophil markers were analyzed in the absence (resting) and presence (activated) of fNLF (10 μM).

**FIGURE 1:**
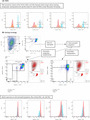
**Approach for flow cytometric analysis**. (**A**) Fluorescence minus one (FMO) controls of the used panel of markers (CD16, CD11b, CD62L, CD10). CD64 was also tested in this panel but is not shown here because it is not discussed further in this article; (**B**) gating strategy for analyzing the blood samples from healthy controls and patients. Flow cytometric analysis was done using FlowJo analysis software (Tree Star Inc.)

Neutrophil subsets were identified by the expression of CD16 and CD62L as described in detail earlier.^30^ Absolute promyelocytes, myelocytes, and metamyelocytes counts were calculated based on CD16/CD11b expression.^31^ An example of the gating and FMO used is shown in Fig. 1. The marker CD64 was used to facilitate differentiation between viral and bacterial infections in suspected COVID-19 patients, as has been demonstrated earlier.^32^ This marker was not used for univariate and bivariate analysis of the neutrophil compartment.

Discriminant analysis of multi-aspect cytometry (DAMACY) fusion analysis was performed as described earlier.^33^^,^^34^ DAMACY first describes the cellular distribution using 2D smoothed histograms of the first two principal components using 100 bins per principal component. The 2D smoothed histograms were created separately for fNLF^−^ and for fNLF^+^ and then fused together. Discriminant analysis was based on the fused histograms using orthogonal projections on latent structures. The DAMACY model was created based on the samples from healthy controls and from COVID-19 critical patients using the first sample measurement and 5-fold crossvalidation with 10 iterations procedure. The bacterial specific marker CD64 was included in the multivariate analysis as it was unknown whether expression of CD64 was of added value in the diagnosis/prognosis of COVID-19 severity. All patients were projected into the model and a mean DAMACY score was calculated.

### Clinical characteristics of suspected COVID-19

Patients who tested SARS-CoV-2 positive by PCR at any point during admission were considered COVID-19 positive. Patients who tested negative on PCR for COVID-19 were assessed in detail for other diagnoses that could explain their clinical condition. Results of bacterial cultures, PCR for other viruses, or another explanation/diagnosis for the symptoms in the letter of discharge were recorded. Patients who were tested PCR negative with no other explaining diagnosis were excluded from analysis.

Patients who tested COVID-19 positive were divided into groups based on disease severity according to the interim guidance of the WHO published on May 27, 2020: Clinical Management of COVID-19.^35^ Moderate disease was defined as clinical signs of mild to moderate pneumonia (including oxygen saturation [SpO_2_] ≥90% in room air). Severe disease was defined as clinical signs of severe pneumonia (including SpO_2_ < 90% in room air) plus one of the following: respiratory rate > 30 breaths/min; severe respiratory distress, but without meeting the criteria for ARDS at any time during admission. Critical disease was defined as severe disease with ARDS at any point during admission. ARDS was defined as SpO_2_/FiO_2_ ≤ 315,^35^ where FiO_2_ is fraction of inspired oxygen.

Baseline characteristics included age, sex, body mass index (BMI), onset of symptoms, ICU admission, death, and duration of admission. Vital functions during admission were collected from the moment FiO_2_ was lowest, according to the Berlin guidelines. The following clinical variables were collected: saturation (%), amount of oxygen received (L/min), type of ventilation, respiratory rate (/min), pulse rate (/min), systolic blood pressure (mmHg), diastolic blood pressure (mmHg), and temperature (°C). Also, laboratory values C-reactive protein (CRP; g/L), total leukocyte count (×10^9^/L), and arterial saturation were retrieved.

### Statistics

Variables are presented as frequencies and percentages for categorical variables and as medians with IQR for continuous variables. Differences in clinical outcomes and demographics between COVID-19 severity groups were assessed with the use of Pearson’s χ^2^ test for categorical data. For continuous data, if multiple groups were compared, a Kruskal-Wallis test was performed and for comparison of two groups, Mann-Whitney *U*-test was used. Control groups for analysis included both healthy controls and COVID-19 PCR negative patients with a proven bacterial or other viral infection. To evaluate differences in neutrophil receptor expression between each of the COVID-19 severity groups and control groups, a Kruskal-Wallis test was applied and post hoc, the Dunn’s test with Bonferonni correction was used to correct for multiple comparisons. GraphPad Prism version 7 (GraphPad software, Inc., San Diego, CA, USA) was used for data visualization. Stata version 13.0 (StataCorp LP, College Station, TX, USA) was used for all statistical analyses. Statistical significance was defined as a *P*-value <0.05.

Multidimensional analysis was performed by application of the DAMACY algorithm as described by us earlier.^33^ The results of all statistical analyses are shown in Supporting Information Table S1.

### Study approval

For this study, a waiver for formal ethical approval was provided by the institutional medical ethics committee under protocol number 20-284/C. The Mini Donor Service received positive approval from the medical ethical committee of UMCU under protocol number 07-125/C. All procedures performed in this study were in accordance with the 1964 Helsinki declaration and its later amendments.

## Results

**FIGURE 2:**
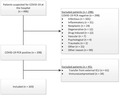
Flow diagram depicting inclusion and exclusion of patients in this study

A total of 496 COVID-19 suspected patients presented to the hospital in the period between March 19, 2020, and May 17, 2020, of whom 198 tested SARS-CoV2 positive by PCR. Of the 298 patients who tested SARS-CoV-2 negative by PCR, 208 patients were diagnosed with a disease other than COVID-19, 17 patients had another viral infection, and 84 patients had a culture-positive bacterial infection (Fig. 2). The microorganisms causing the viral and bacterial infections and the accompanying diagnoses are identified in Supporting Information Table S2.

A total of 95 COVID-19-positive patients were excluded (transfer from an external ICU or immunocompromised). The baseline characteristics of the immunocompromised patients excluded from the study are described in Supporting Information Table S3. Of the included patients 10 patients had moderate disease, 16 patients were diagnosed with severe disease, and 77 patients were classified as critically ill. None of the patients with moderate disease were admitted to the hospital. Baseline characteristics of the included patients are shown in Table 1. Relevant medication use of the included COVID-19-positive patients is described in Supporting Information Table S4.

Overall, there were no significant differences in gender, age, BMI, and onset of symptoms among the COVID-19-positive patients based on disease severity. However, there was a trend toward a higher median age in the critical group. Clinically, patients with more severe disease at presentation at the emergency department required a higher FiO_2_ and showed enhanced respiratory rate and CRP levels. ICU admission was required for 36% of the patients with critical disease, and 27% died during hospital admission.

**FIGURE 3:**
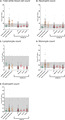
**Absolute cell counts of white blood cell populations in the ER**. Absolute cell counts of (**A**) Total white blood cells; (**B**) Neutrophils; (**C**) Lymphocytes; (**D**); Monocytes and (**E**) Eosinophils in peripheral blood of healthy controls (HC, *n* = 23), patients with other diagnoses categorized as bacterial (*n* = 84) or viral (*n* = 17) and coronavirus disease 2019 (COVID-19) patients as a total group (*n* = 103) and categorized according to the WHO classification system for COVID-19 as moderate (*n* = 10), severe (*n* = 16), or critical (*n* = 77) disease. The normal range of cell counts is displayed by the gray area. Results of the statistical analysis are shown in Supporting Information Table 1.1. Data are represented as individual dots with medians and interquartile range (IQR)

**FIGURE 4:**
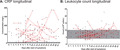
Plasma concentrations of (**A**) C-reactive protein (CRP) and (**B**) leukocytes over time after the onset of symptoms in coronavirus disease 2019 (COVID-19) patients (*n* = 78) with critical or moderate disease. The normal range of cell counts is displayed by the gray area. Data are represented as individual black dots. If multiple measurements were done for one patient, different measurements are connected with a red line to indicate paired data. Data were retrieved from the patients’ records

Of the sampled patients, most met the ARDS criteria at the time of blood collection (73%) and almost all developed respiratory distress within 3 d after blood was drawn (97%). The total white blood cell count, granulocyte count, neutrophil count, eosinophil count, and monocyte count did not differ between the COVID-19-positive patients based on disease severity. However, the lymphocyte counts decreased with disease severity; the lymphocyte count in the moderate group was significantly different compared to the critical disease group. Cell counts are displayed in Fig. 3. Corresponding statistics can be found in the Supporting Information Table S1.1. The longitudinal data regarding CRP levels and total leukocyte counts show that these inflammation-associated markers were stable over time (see Fig. 4).

### No signs of hyperinflammation in the neutrophil compartment during COVID-19 at hospital presentation

**FIGURE 5:**
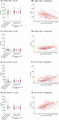
Neutrophil activation markers CD62L and CD11b in neutrophils in the absence and presence of a bacterial stimulus (N-Formyl-norleucyl-leucyl-phenylalanine [fNLF], 10 μM) to induce activation, as measured in the ER and over time (longitudinal). Flow cytometric measurements were done in the ER using peripheral blood from patients with other diagnoses categorized as bacterial (*n* = 84) or viral (*n* = 17) infections and from coronavirus disease 2019 (COVID-19) patients as a total group (*n* = 103) and categorized according to the WHO classification system for COVID-19 as moderate (*n* = 10), severe (*n* = 16), or critical (*n* = 77) disease. A group of healthy controls (*n* = 23) was also included. Longitudinal measurements were done with peripheral blood from patients on the COVID-19 ward (*n* = 78) who were categorized as having moderate or critical disease. For the majority of patients multiple measurements were done overtime. (**A**) CD62L without fNLF in the ER; (**B**) CD62L without fNLF longitudinal; (**C**) CD62L with fNLF in the ER; (**D**) CD62L with fNLF longitudinal; (**E**) CD11b without fNLF in the ER; (**F**) CD11b without fNLF longitudinal; (**G**) CD11b with fNLF in the ER; and (**H**) CD11b with fNLF longitudinal. Results of the statistical analysis are shown in Supporting Information Table S1.2. Data are represented as individual dots with medians and interquartile range (IQR; ER samples) or as individual data points connected with a red line to indicate paired data (longitudinal data)

In order to investigate the involvement of a hyperactivated neutrophil compartment in COVID-19 we compared the neutrophil activation markers CD11b and CD62L between healthy controls and COVD-19-positive patients based on disease severity. In the peripheral blood, the expression of CD11b and CD62L on neutrophils did not significantly differ between the COVID-19 severity groups at hospital presentation (see Fig. 5A, C, E, G). Remarkably, expression of CD11b and CD62L was also not higher in COVID-19 patients when compared to healthy controls (Fig. 5). Furthermore, CD11b was significantly lower in patients with bacterial infections, other viral infections and in the combined COVID-19 group compared to healthy controls (see Fig. 5 and Supporting Information Table S1.2). To examine the activation status of the neutrophils at hospital presentation or their ability to undergo activation, we investigated the responsiveness of the neutrophils to the bacterial stimulus fNLF. Neutrophils from all COVID-19 patients as well as patients with bacterial or viral infections were characterized by a significantly lower responsiveness to fNLF compared to healthy controls based on CD11b expression.

The longitudinal data supported the finding that no clear activation of neutrophils was evident at presentation in the hospital as the expression of CD11b was relatively low at this early time point (Fig. 5F), whereas CD62L was high (Fig. 5B). Interestingly, during admission the neutrophils acquired a more activated phenotype characterized by a trend to a higher expression of CD11b and a lower expression of CD62L.

The unexpected low levels of ex vivo activation markers measured in both COVID-19 patients and the other patient groups prompted us to test our automatic flow method in an experimental set-up of acute inflammation. The same flow protocol was applied to healthy control individuals participating in an experimental endotoxemia trial. As can be seen in Supporting Information Fig. S1, acute systemic inflammation present 3 h after LPS challenge leads to up-regulation of CD11b when compared to prechallenge levels. This indicates that CD11b expression can be induced in vivo and, importantly, fully automated flow cytometry can measure neutrophil activation in vivo.

### Association of COVID-19 with the presence of young mature neutrophils in the peripheral blood

**FIGURE 6:**
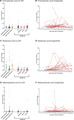
**Absolute cell counts of neutrophil progenitors gated based on CD11b/CD16 expression as measured in the ER and over time (longitudinal)**. Flow cytometric measurements were done in the ER using peripheral blood from patients with other diagnoses categorized as bacterial (*n* = 84) or viral (*n* = 17) infections and coronavirus disease 2019 (COVID-19) patients as a total group (*n* = 103) and categorized according to the WHO classification system for COVID-19 as moderate (*n* = 10), severe (*n* = 16), or critical (*n* = 77) disease. A group of healthy controls (*n* = 23) was also included. Longitudinal measurements were done with peripheral blood from patients on the COVID-19 ward (*n* = 78) who were categorized as having moderate or critical disease. For the majority of patients multiple measurements were done overtime. (**A**) Promyelocyte counts in the ER; (**B**) promyelocyte counts longitudinal; (**C**) myelocyte counts on the ER; (**D**) myelocyte counts longitudinal; (**E**) metamyelocyte counts on the ER; and (**F**) metamyelocyte counts longitudinal. Results of the statistical analysis are shown in Supporting Information Table S1.3. Data are represented as individual dots with medians and interquartile range (IQR; ER samples) or as individual data points connected with a red line to indicate paired data (longitudinal data)

Acute inflammatory diseases are normally associated with the presence of young neutrophils (banded cells) and neutrophil progenitors in the peripheral blood.^30^^,^^36^^,^^37^ However in COVID-19 patients, the absolute number of promyelocytes in the blood of COVID-19 patients did not significantly differ from the number in healthy controls (Fig. 6A). The absolute number of myelocytes was significantly lower in all COVID-19 groups compared to healthy controls as can be seen in Fig. 6C. The number of metamyelocytes increased slightly with disease severity in COVID-19 patients with a significant difference noted between the moderate and critical groups (Fig. 6E and Supporting Information Table S1.3). However, the amount of metamyelocytes was still relatively low in the COVID-19 group compared to patients with bacterial infections.

The longitudinal data show normalization of the number of neutrophil progenitors with time as the numbers become similar to those found in healthy control individuals (Fig. 6B, D, F).

**FIGURE 7:**
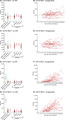
**Expression of neutrophil maturation markers CD16 and CD10 in the absence and presence of a bacterial stimulus (N-Formyl-norleucyl-leucyl-phenylalanine [fNLF], 10 μM) to induce activation, as measured in the ER and over time (longitudinal)**. Flow cytometric measurements were done in the ER using peripheral blood from patients with other diagnoses categorized as bacterial (*n* = 84) or viral (*n* = 17) infections and coronavirus disease 2019 (COVID-19) patients as a total group (*n* = 103) and categorized according to the WHO classification system for COVID-19 as moderate (*n* = 10), severe (*n* = 16), or critical (*n* = 77) disease. A group of healthy controls (*n* = 23) was also included. Longitudinal measurements were done with peripheral blood from patients on the COVID-19 ward (*n* = 78) who were categorized as having moderate or critical disease. For the majority of patients multiple measurements were done overtime. (**A**) CD16 without fNLF in the ER; (**B**) CD16 without fNLF longitudinal; (**C**) CD16 with fNLF in the ER; (**D**) CD16 with fNLF longitudinal; (**E**) CD10 without fNLF in the ER; (**F**) CD10 without fNLF longitudinal; (**G**) CD10 with fNLF in the ER; and (**H**) CD10 with fNLF longitudinal. Results of the statistical analysis are shown in Supporting Information Table S1.4. Data are represented as individual dots with medians and interquartile range (IQR; ER samples) or as individual data points connected with a red line to indicate paired data (longitudinal data)

Furthermore, these findings coincided with a significantly lower expression of the neutrophil maturation marker FcγRIII (CD16) on the total granulocyte population in COVID-19 patients, decreasing with higher disease severity, suggesting a neutrophil compartment that is younger than normal (Fig. 7A). CD16 expression in the group with bacterial infections was even lower than the expression in the COVID-19 group, indicating that the peripheral neutrophil pool consists of even younger neutrophils in bacterial infections. The neutrophil maturation marker CD10 was significantly lower in “all” COVID-19 severity groups (*P* < 0.02 for all comparisons, Supporting Information Table S1.4) compared to controls. However, patients with other bacterial and viral infections showed a similar large decrease in CD10. Even after exposure to fNLF, the expression of CD10 by neutrophils from COVID-19 and other disease groups stays significantly lower compared to healthy controls (Fig. 7G). The longitudinal data of CD16 indicated that expression of this marker slightly increased over time in some patients, but generally stayed in the low range (see Fig. 7B).

Surprisingly, CD10 expression was low during the early days after onset of COVID-19 symptoms and continued to stay low after stimulation with fNLF (Fig. 7F, H)). CD10 expression generally seemed to normalize with time, although values remained relatively low compared to healthy controls.

### Multidimensional analysis of the complete dataset

**FIGURE 8:**
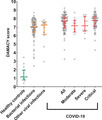
**Multidimensional analysis of neutrophil characteristics in coronavirus disease 2019 (COVID-19) patients compared to controls**. Based on the differences in neutrophil characteristics in the presence and absence of N-Formyl-norleucyl-leucyl-phenylalanine (fNLF; 10 μM) present in COVID-19 patients compared to controls, a discriminant analysis of multi-aspect cytometry (DAMACY) score is calculated. A higher score indicated more deviations in neutrophil marker expression compared to healthy controls. DAMACY scores are shown for healthy controls (*n* = 23), patients with other diagnoses categorized as bacterial (*n* = 84) or viral (*n* = 17) and COVID-19 patients as a total group (*n* = 103) and categorized according to the WHO classification system for COVID-19 as moderate (*n* = 10), severe (*n* = 16), or critical (*n* = 77) disease

In data shown thus far, the analysis was one and 2D and was carried out by multiple gating. This approach might have missed correlations that were not expected. Therefore, we analyzed the complete dataset by the algorithm DAMACY^34^ that allows comparison of flow cytometric profiles between individual patients. An algorithm was used in the DAMACY analysis to extract putative COVID-19 or other disease-specific cellular differences when compared to healthy controls. Based on these differences a DAMACY score was calculated. A higher score indicated more deviation from healthy control samples. The DAMACY score clearly discriminated between healthy controls and infectious diseases including COVID-19 (Fig. 8 and Supporting Information Table S1.5). However, no significant differences were found between severities of COVID-19 or between COVID-19 and other infectious diseases. The changes seen in neutrophil marker expression were therefore not COVID-19 specific, but indicative of the presence of disease in general.

### Maturation dissociation in systemic infectious disease

Despite the fact that the overall expression of CD16 was decreased in COVID-19 patients, no clear CD16^dim^/CD62L^bright^ subset was found in these patients suggesting a low number of banded cells in the blood of these patients. This is in marked contrast to acute inflammatory conditions such as noted during experimental endotoxemia^38–40^ and multitrauma.^41–43^ A low number of banded cells was confirmed by cytospin analysis (see Supporting Information Fig. S2). In line, no CD16^dim^/CD10^dim^ immature neutrophils (recent bone marrow emigrants^40^) were found in COVID-19 patients, again supporting the low numbers of banded cells in the peripheral blood.^44^

## Discussion

Several landmark studies^6^^,^^20^^,^^21^^,^^45^ suggest that the pathogenesis of critical COVID-19 is mediated by hyperinflammatory processes that can lead to ARDS and death. As neutrophils are thought to be main effector cells in classical ARDS, some studies aimed to investigate the neutrophil involvement in patients with suspected COVID-19.^16^ Until now no clear evidence has been published on hyperactivation of the neutrophil compartment in COVID-19 patients at hospital presentation, before ICU admission. In fact, a recent study applying whole blood transcriptomics shows signatures for the presence of immature neutrophils without signs of hyperinflammation.^46^ Furthermore, post-mortem analysis demonstrated hardly any influx of neutrophils in the pulmonary tissue, questioning the pathogenesis of the ARDS-like clinical presentation seen in COVID-19 patients.^47^ Our study tested the hypothesis that the innate immune response during the first 3 d of hospital admission was characterized by hyperinflammation. COVID-19 patients showed a maturation dissociation characterized by a low CD10/CD16 expression of all blood neutrophils, even on cells with no banded nucleus. This coincided with a low expression of activation markers, which argued against activation of the neutrophil compartment at the time point of clinical admission. Much to our surprise, patients with other types of infection exhibited a similarly low neutrophil activation signature on hospital presentation. This might be explained by the fact that our new, automated and fast method to measure neutrophil activation markers does not detect artificial activation during presence in the blood collection tube.^28^ In our previous research we showed a specific activation of neutrophils during prolonged (>30 min) presence in a blood tube.^28^ The sensitivity of this artificial activation might be more prominent for cells that are primed by disease in vivo. This situation is completely different during acute inflammation associated with the presence of banded neutrophils such as that found after LPS challenge^30^ and during severe trauma.^10^ During acute inflammation a clear induction of CD11b expression was found^10^ (also see Supporting Information Fig. S1). It is tempting to speculate that the difference between acute and more chronic inflammation might be caused by homing of activated cells to the tissues.^48^^,^^49^ Our longitudinal data are also suggestive of this. Upon presentation at the ER, neutrophils from COVID-19 patients were characterized by a young (CD10^dim^/CD16^dim^) population with a low activation phenotype (CD11b^dim^/CD62L^high^). It is tempting to speculate that activated mature cells may have migrated to the tissues leaving behind relatively young nonactivated neutrophils, a situation also found in trauma patients.^49^ Interestingly, this situation seemed to normalize with time as the patients recovered, leading to a counterintuitive activation of the neutrophil compartment characterized by a rise in CD11b expression and a lower expression of CD62L (Fig. 5B, F). Also, an increase in cellular age was detected (higher CD10 expression). The apparent increase in myelocyte numbers over time in the peripheral blood of COVID-19 patients (Fig. 6D) is supportive of the activation of neutrophilopoiesis in the bone marrow. These data also imply that an acute inflammatory event may have occurred prior to admittance to the hospital. This hypothesis has yet to be tested in a new study on mild COVID-19 patients.

The approach employed in this study also led to the surprising finding that the pathogenesis of COVID-19 before admission to the ICU is characterized by the occurrence of CD10 low cells in the absence of increased activation markers on neutrophils. This seems in marked contrast to the literature suggesting the presence of hyperinflammation in COVID-19 patients that is associated with a cytokine storm, resulting in excessive innate immune activation. These seemingly contradictory findings can be explained by various published results. First of all, several studies describing evidence of hyperinflammation in COVID-19 pooled their pre- and post-ICU data. Data post-ICU are compromised by tissue damage associated with ventilation and processes associated with ischemia and reperfusion injury caused by thromboembolic events associated with COVID-19. In addition, data are influenced by the treatment received in the ICU such as invasive ventilation, central catheters, dialysis, and inotropic support. Neutrophils are known to become systemically activated under these conditions.^50^^,^^51^ Secondly, neutrophils become primed during systemic inflammatory disease, causing them to be hyper-responsive for ex vivo manipulation resulting in high artificial expression of activation markers. Our flow method is much less sensitive to these ex vivo artifacts compared to most flow cytometric methods used in other articles.^28^ Thirdly, the data on systemic cytokines in COVID-19 are not unambiguously supportive for a cytokine storm leading to systemic activation of innate immune cells.^6^^,^^20^^,^^52^^,^^53^ Significant decreases in neutrophil maturation markers were seen (CD10 and CD16) in COVID-19 patients in the complete neutrophil compartment in the peripheral blood. Remarkably, no increase in the number of CD16^dim^ banded cells was detected such as that found under conditions of acute inflammation.^38^^,^^40–42^ This indicated that the overall neutrophil compartment was younger and that the decrease in maturation markers was not due to the mobilization of a specific neutrophil subset into the blood but rather a shift of the total population. This hypothesis was supported by the finding that all neutrophils in all COVID-19 patients were very low in expression of CD10 irrespective of disease severity. It is important to emphasize that the CD10 protein is also present on the inside of the cells as it is up-regulated by activation by a formyl-peptide, albeit significantly lower in COVID-19 and other diseases than in neutrophils of healthy controls (Fig. 7E and 7G). This indicates that CD10 is probably present in low amounts (on the surface and in storage pools) in blood neutrophils during COVID-19 and other infectious diseases, possibly because of an altered or accelerated maturation process. Because the healthy controls are relatively young compared to the patients included in this study, it is important to emphasize that the expression of nCD10/neprilysin on neutrophils measured by automated flow cytometry is not dependent on the age of the donor.^28^^,^^59^

Expression of CD10/neprilysin is high as seen in neutrophils in healthy control blood (Fig. 7E and Marini et al.^54^). Interestingly, the low expression of neutrophil CD10 is already present at the start of the COVID-19 symptoms (Fig. 7F). As we have only sampled COVID-19 patients who presented in the hospital, the possibility exists that low CD10 expression during onset of the disease is associated with deterioration and hospitalization. It can possibly be used as a discriminator between more benign disease course and deterioration of disease. It is, therefore, critical to study neutrophil CD10 expression in a future cohort of COVID-19 patients positive for SARS-CoV-2 by PCR who are asymptomatic or have mild disease.

Based on our findings and conflicting studies in literature, hyperinflammation does not seem to be a dominant feature found in symptomatic COVID-19 patients at hospital presentation. This view is supported by a recent meta-analysis that leads to the conclusion that the pathogenesis of COVID-19 is not mediated by a significant cytokine storm.^60^ However, it could be that hyperinflammation is the consequence of severe/critical illness in general and ICU admission. This might also lead to local activation of neutrophils and induction of netosis in the lung.^61^^,^^62^ Pulmonary edema and thromboembolic complications seem to be the main reason for clinical deterioration of disease in SARS-CoV-2-infected patients. It has been suggested that the low expression of the ACE2 receptor caused by viral entry in ACE2^+^ cells^55^ is involved in both COVID-19-related hypercoagulopathy and pulmonary edema. Less available ACE2 on the cell surface due to consumption by viral entry and thus lower inactivation of bradykinin1-9 may result in increased bradykinin1-9 plasma concentrations, subsequently causing vascular leakage, angioedema, and inflammation.^53^ In addition, lack of ACE2 might be involved in the thrombotic complications observed in COVID-19 patients.^22^ In parallel with the ACE2-mediated breakdown, bradykinin1-9 can also be metabolized by CD10 (neprilysin),^24^ which is a well-known maturation and activation marker expressed by neutrophils.^56^ Here, we speculate that the low expression of CD10 on neutrophils obtained during severe systemic disease, might be of particular relevance for COVID-19 patients as the airways are specifically devoid of ACE2 expression caused by viral entry.^57^^,^^58^ In this situation CD10 on neutrophils might become rate-limiting for sufficient inactivation of bradykinin, whereas during “normal” inflammatory diseases, ACE2 can take over. In this study, we showed a clear decrease in the CD10 receptor on neutrophils in all COVID-19 severity groups that are admitted to the hospital, indicating that decreased CD10 on neutrophils might be involved in the compromised bradykinin pathway in COVID-19 patients.

This study was limited by the inability to obtain blood from age-matched healthy controls due to the restrictions imposed by COVID-19. It could be that some of the data we have shown are affected by difference in age between the study groups. However, we have investigated similar neutrophil activation markers in previous studies and found no difference in baseline expression levels between ages.^59^ Furthermore, we state that there is no neutrophil activation in COVID-19 patients at presentation. This study entails neutrophil activation measured only in the peripheral blood with limited number of markers. It is possible that neutrophils are activated in the tissues (e.g., lung tissue), which cannot be investigated without invasive lung/tissue biopsies. It could also be that there was neutrophil activation in the peripheral blood, but that this did not result in neutrophil surface receptor (CD11b and CD62L) changes. This study also did not investigate the possible role of netosis in COVID-19 patients and the disease pathology, a concept that has been put forward in COVID-19 literature.^61^^,^^62^

The putative absence of hyperinflammation in COVID-19 patients is important in the understanding of the pathophysiology of COVID-19 and possible treatment options. No trials focused on specific inhibition of inflammation have shown efficacy in the treatment of COVID-19 patients outside the ICU.^21^ Candidates for the treatment of COVID-19 should therefore aim to prevent pulmonary edema and/or thromboembolic complications. The success of dexamethasone treatment in oxygen-dependent COVID-19 patients might be explained by its clear vasoconstrictive effects, in addition to its anti-inflammatory effects.^63^ Assuming the therapeutic effect of the vasoconstrictive ability of dexamethasone, additional therapeutic opportunities might still lie in influencing the neutrophil compartment, as the CD10 expression on neutrophils is possibly important in the pathophysiology of severe COVID-19 disease.

## Conclusion

COVID-19 severity is associated with a maturation dissociation in the neutrophil compartment, characterized by a release into the peripheral blood of neutrophils with a low CD16 and CD10 expression. The younger neutrophils and the lack of expression of the CD10 receptor might be involved in the compromised bradykinin pathway in COVID-19 patients. However, neutrophil cell counts are not increased by a SARS-CoV-2 infection and no signs of increased activation nor hyperinflammation is demonstrated in the neutrophil compartment in the peripheral blood at hospital admission. This indicates that hyperinflammation, determined by leukocyte (progenitor) numbers and the expression of neutrophil activation markers, is not present at hospital admission of COVID-19 patients.

## Authorship

R.S. and S.H.B. are both stated as first authors. The authorship order is assigned based on coordination of the study, the process of interpretation of writing, and submitting of the manuscript. R.S.: Coordination of construction of the clinical database, data analysis, data interpretation, and writing of the manuscript; S.H.B.: Coordination of sample processing and analysis, data analysis, data interpretation, and writing of the manuscript; B.J.J.B.: Construction of the clinical database, data analysis, data interpretation, and writing of the manuscript; G.H.T.: Data analysis and multi-dimensional analysis; G.G.: Sample processing and analysis, data analysis, and comments on final version of the paper; N.K.N.J.: Construction of the clinical database, data analysis, and data interpretation; W.B.: Data analysis and data interpretation; D.E.J.S.: Data analysis and data interpretation; E.M.D.: Sample processing and analysis, conceptual advice, and comments on final version of the paper; S.N.: Conceptual advice and comments on final version of the paper; H.M.R.G.: Conceptual advice and comments on final version of the paper; N.V.: Conceptual advice and comments on final version of the paper; J.J.J.: Data analysis and multidimensional analysis; F.H.: Design of the study, conceptual advice, and comments on final version of the paper; L.P.H.L.: Design of the study, conceptual advice, and comments on final version of the paper; K.A.H.K.: Design of the study, conceptual advice, and comments on final version of the paper; and L.K.: Design and organization of the study, interpretation, conceptual advice, and comments on final version of the paper.

## Supplementary Material

jlb10860-sup-0001-tableS1 Table S1

jlb10860-sup-0002-tableS2 Table S2

jlb10860-sup-0003-tableS3 Table S3

jlb10860-sup-0004-tableS4 Table S4

jlb10860-sup-0005-figureS1 Figure S1

jlb10860-sup-0006-figureS2 Figure S2

## Abbreviations

ACE2: Angiotensin-converting enzyme 2
ARDS: Acute respiratory distress syndrome
BMI: Body mass index
CD: Cluster of differentiation
COVID-19: Coronavirus disease 2019
DAMACY: Discriminant analysis of multi-aspect cytometry
FiO_2_: Fraction of inspired oxygen
FMO: Fluorescence minus one
fNLF: N-Formyl-norleucyl-leucyl-phenylalanine
ICU: Intensive care unit
IQR: Interquartile range
SARS-CoV-2: Severe acute respiratory syndrome coronavirus 2
SpO_2_: Oxygen saturation
