# Supplementary material for: Flow cytometric evaluation of the neutrophil compartment in COVID-19 at hospital presentation: A normal response to an abnormal situation
Source: J Leukoc Biol. 2020 Dec 22;109(1):99–114. doi: 10.1002/JLB.5COVA0820-520RRR (PMC10016865; doi:10.1002/JLB.5COVA0820-520RRR)
Supplement: jlb10860-sup-0001-tableS1 — Table S1 [file jlb10860-sup-0001-tables1.docx]

**Supplemental Table S1**

**S1.1:** Statistical analysis of cell counts as measured on hospital presentation. Results correspond to the results shown in figure 3 in the main article. Kruskal-Wallis tests were carried out followed by a post-hoc Dunn’s test with Bonferroni correction for multiple comparison. Significance was defined as P < 0.05 (significant results are also shown in bold digits). The panels on the left show the results of the COVID-19 patients subdivided according to disease severity class, compared to healthy controls (HC), patients with bacterial infections and other viral infections. The panels on the right show the results of a separate analysis of the COVID-19 group as a whole compared to healthy controls, patients with bacterial infections and other viral infections.

**S1.2:** Statistical analysis of expression of neutrophil activation markers CD62L and CD11b, with or without fNLF stimulation as measured on hospital presentation. Results correspond to the results shown in Figure 5 in the main article. Kruskal-Wallis tests were carried out followed by a post-hoc Dunn’s test with Bonferroni correction for multiple comparison. Significance was defined as P < 0.05 (significant results are also shown in bold digits). The panels on the left show the results of the COVID-19 patients subdivided according to disease severity class, compared to healthy controls (HC), patients with bacterial infections and other viral infections. The panels on the right show the results of a separate analysis of the COVID-19 group as a whole compared to healthy controls, patients with bacterial infections and other viral infections.

**S1.3:** Statistical analysis of neutrophil progenitor counts as based on CD11b/CD16 gating of the granulocyte population, as measured on hospital presentation. Results correspond to the results shown in Figure 6 in the main article. Kruskal-Wallis tests were carried out followed by a post-hoc Dunn’s test with Bonferroni correction for multiple comparison. Significance was defined as P < 0.05 (significant results are also shown in bold digits). The panels on the left show the results of the COVID-19 patients subdivided according to disease severity class, compared to healthy controls (HC), patients with bacterial infections and other viral infections. The panels on the right show the results of a separate analysis of the COVID-19 group as a whole compared to healthy controls, patients with bacterial infections and other viral infections.

**S1.4:** Statistical analysis of neutrophil maturation markers CD16 and CD10, with or without fNLF stimulation as measured on hospital presentation. Results correspond to the results shown in Figure 7 in the main article. Kruskal-Wallis tests were carried out followed by a post-hoc Dunn’s test with Bonferroni correction for multiple correction. Significance was defined as P < 0.05 (significant results are also shown in bold digits). The panels on the left show the results of the COVID-19 patients subdivided according to disease severity class, compared to healthy controls (HC), patients with bacterial infections and other viral infections. The panels on the right show the results of a separate analysis of the COVID-19 group as a whole compared to healthy controls, patients with bacterial infections and other viral infections.

**S1.5:** Statistical analysis of the DAMACY score, as calculated based on the data retrieved on hospital presentation. Results correspond to the results shown in Figure 8 in the main article. Kruskal-Wallis tests were done followed by a post-hoc Dunn’s test with Bonferroni correction for multiple comparison. Significance was defined as P < 0.05 (significant results are also shown in bold digits). The panels on the left show the results of the COVID-19 patients subdivided according to disease severity class, compared to healthy controls (HC), patients with bacterial infections and other viral infections. The panels on the right show the results of a separate analysis of the COVID-19 group as a whole compared to healthy controls, patients with bacterial infections and other viral infections.
