# Supplementary material for: Flow cytometric evaluation of the neutrophil compartment in COVID-19 at hospital presentation: A normal response to an abnormal situation
Source: J Leukoc Biol. 2020 Dec 22;109(1):99–114. doi: 10.1002/JLB.5COVA0820-520RRR (PMC10016865; doi:10.1002/JLB.5COVA0820-520RRR)
Supplement: jlb10860-sup-0002-tableS2 — Table S2 [file jlb10860-sup-0002-tables2.docx]

**Supplemental Table S2:** Specification and quantification of the infections scored in the “Bacterial infections” and “Viral infections” groups that were compared to the COVID-19 patients as disease controls. The type of infection is shown with the pathogen that was cultured. If no pathogen was cultured, if results were inconclusive or if the results could not be retrieved from the patient history, data was scored as unknown.
