# Supplementary material for: Flow cytometric evaluation of the neutrophil compartment in COVID-19 at hospital presentation: A normal response to an abnormal situation
Source: J Leukoc Biol. 2020 Dec 22;109(1):99–114. doi: 10.1002/JLB.5COVA0820-520RRR (PMC10016865; doi:10.1002/JLB.5COVA0820-520RRR)
Supplement: jlb10860-sup-0003-tableS3 — Table S3 [file jlb10860-sup-0003-tables3.docx]

**Supplemental Table S3:** Baseline characteristics of excluded immunocompromised COVID-19 patients shown along with included COVID-19 patients according to disease severity.
