# Supplementary material for: Flow cytometric evaluation of the neutrophil compartment in COVID-19 at hospital presentation: A normal response to an abnormal situation
Source: J Leukoc Biol. 2020 Dec 22;109(1):99–114. doi: 10.1002/JLB.5COVA0820-520RRR (PMC10016865; doi:10.1002/JLB.5COVA0820-520RRR)
Supplement: jlb10860-sup-0004-tableS4 — Table S4 [file jlb10860-sup-0004-tables4.docx]

**Supplemental Table S4:** Use of medications in COVID-19 patients (N = 103) included in the study. Medications prescribed during treatment in the hospital and those used by included patients prior to hospital admission are shown.
