# Supplementary material for: Flow cytometric evaluation of the neutrophil compartment in COVID-19 at hospital presentation: A normal response to an abnormal situation
Source: J Leukoc Biol. 2020 Dec 22;109(1):99–114. doi: 10.1002/JLB.5COVA0820-520RRR (PMC10016865; doi:10.1002/JLB.5COVA0820-520RRR)
Supplement: jlb10860-sup-0005-figureS1 — Figure S1 [file jlb10860-sup-0005-figures1.docx]

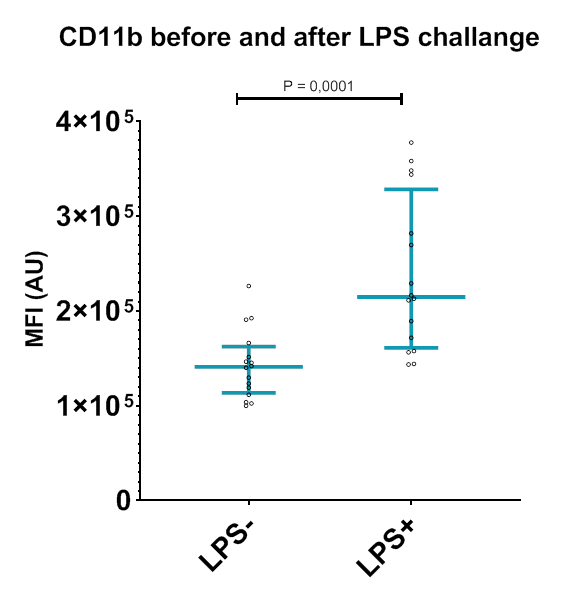


**Supplemental Figure S1**: CD11b expression on neutrophils as measured by the AQUIOS CL^®^ “load & go” flow cytometer before (LPS-) and 3 hours after (LPS+) in vivo LPS administration to 18 healthy volunteers. A two-tailed Mann-Whitney test showed a P = 0.0001 when comparing LPS- to LPS+.
