# Supplementary material for: Flow cytometric evaluation of the neutrophil compartment in COVID-19 at hospital presentation: A normal response to an abnormal situation
Source: J Leukoc Biol. 2020 Dec 22;109(1):99–114. doi: 10.1002/JLB.5COVA0820-520RRR (PMC10016865; doi:10.1002/JLB.5COVA0820-520RRR)
Supplement: jlb10860-sup-0006-figureS2 — Figure S2 [file jlb10860-sup-0006-figures2.docx]

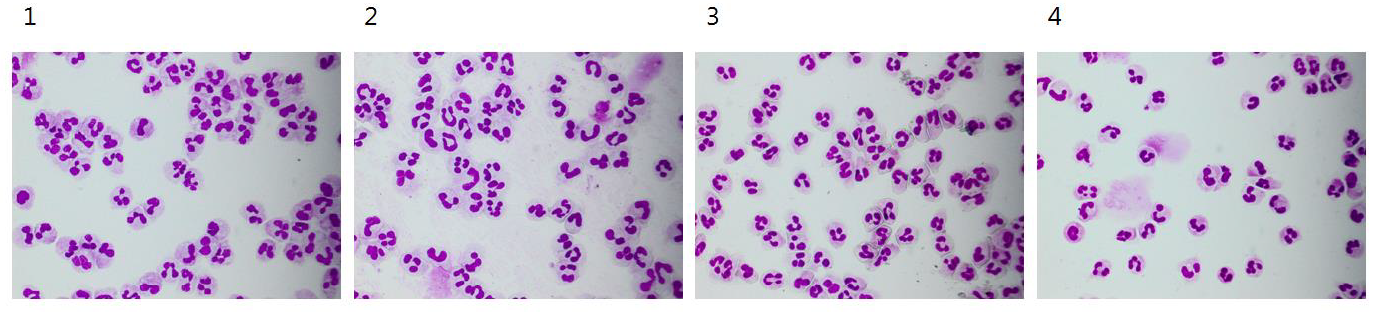
**Supplemental Figure S2:** Morphology of sorted neutrophils with an overall low CD10 expression in the CD16bright/CD62Lbright population,. These neutrophils exhibit normal mature characteristics with only few neutrophils with a banded nucleus (within normal ranges). The cell preparations are from four (1-4) randomly chosen different COVID-19 patients.
